# Coastal evacuations by fish during extreme weather events

**DOI:** 10.1038/srep30280

**Published:** 2016-07-26

**Authors:** Helen Bailey, David H. Secor

**Affiliations:** 1 Chesapeake Biological Laboratory, University of Maryland Center for Environmental Science, P.O. Box 38, Solomons, MD 20688, USA

## Abstract

An increase in the intensity and frequency of extreme events is predicted to occur as a result of climate change. In coastal ecosystems, hurricanes and flooding can cause dramatic changes in water quality resulting in large mortality events in estuarine fauna. Facultative migration behaviors represent a key adaptation by which animals can evacuate ecological catastrophes, but remain poorly studied in marine systems. Here we identify coastal evacuations by otherwise resident riverine striped bass in the Hudson River Estuary, New York, USA, caused by an intense period of tropical storms in autumn 2011. These storms produced record rainfall and high water discharges into the Hudson River Estuary that increased the water level and reduced the water temperature, salinity and dissolved oxygen levels. Striped bass moved out of the estuary, exhibiting novel migration behaviours, that may have been in response to the strong flow and unsuitable conditions. In the months following the storms, some fish demonstrated exploratory trips back to the estuary, which may have been to assess the conditions before returning for the remainder of the winter. Behavioural adaptions to weather events by striped bass and other coastal fishes will depend on maintenance of key population segments and unimpeded evacuation routes.

Climate extremes have important impacts on society and ecosystems^1^,^2^,^3^. These extreme events are by definition rare, although they may become more common in the future as a result of climate change^4^. Evacuations are a principal means by which humans and animals react to ecological catastrophes, but behavioural and environmental impediments can intervene. Populations and communities that are less mobile or obligatory in their migration behaviours are more vulnerable than those that can conditionally respond to extreme events. Among the most migratory animals, birds and fishes, evacuations likely represent a central adaptation to frequent events, but remain poorly documented. The majority of these studies have been on terrestrial ecosystems^1^,^5^ and there are far fewer examples for the aquatic environment^6^,^7^. Studying fish and bird evacuations to weather events has largely relied on serendipity, which precludes strong design elements owing to investigator hazards, unpredictability in timing, and exclusion of experimental controls.

Coastal ecosystems, which receive large inputs from upstream watersheds, are particularly vulnerable to catastrophic change due to hurricanes and other storm events. Sudden delivery of upstream nutrients and sediments and resultant increases in system respiration and hypoxia can result in large mortalities in estuarine fauna that cannot evade such events^8^. How fishes evade such events remains unknown.

Studies of storm events have indicated that sharks and sea snakes can detect changes in barometric pressure associated with the approach of a storm and they respond by taking refuge^9^,^10^. The American lobster (*Homarus americanus*) exhibited increased movement down an estuary towards the coast in association with a storm event^11^. Movements of teleost fish^12^ and elasmobranchs^13^ similarly support the hypothesis that storms can induce movements and shifts in distribution. This in turn alters food webs. For example, changes in reproductive rates and foraging behaviour of marine mammals were observed after a storm, likely in response to changes in prey distribution^14^,^15^.

In many cases our understanding of the effect of extreme events on aquatic organisms has been based on surveys before and after the event. However, relevant ecological responses that are likely behavioural and dynamic, are coarsely represented in surveyed changes in spatial density. Characterizing the movements of highly mobile species during these events provides important information on their behavioural response and may help to predict and mitigate the impacts of future and more frequent events with climate change^13^. Telemetry has been used to determine the habitat use of aquatic species^16^, but only rarely to record detailed movements during extreme events^13^,^17^.

Here, our objective was to determine the response of a key predatory fish species, striped bass (*Morone saxtilis*), to two tropical storm events by examining their individual movements using acoustic telemetry and a before-during-after-control-impact approach. Partial migration occurs in this species: seasonal migrations that persist throughout the lifespan, but differ between population components (aka contingents)^18^. Overlapping migration pathways by contingents occur in the Hudson River Estuary, which harbours one of the largest populations of this species^19^,^20^,^21^. The upper estuary (UEC) and lower estuary contingents (LEC) occupy the estuary throughout most of the year, but with some movement into the surrounding coastal areas during the winter months^19^. In 2011, there were two extreme events beginning with Hurricane Irene, which had weakened to a tropical storm by the time it made landfall at New York City (28^th^ August). This was followed just over a week later by Tropical Storm Lee (6^th^–7^th^ September). How these striped bass contingents respond to extreme events was the focus of this study. We also analyzed survey data for white perch (*Morone americana*) to determine if a similar pattern was detected for another coastal fish species. White perch occur year-round throughout the Hudson River, but are less abundant in the lower estuary^22^,^23^. Although the survey data do not provide information on individual movements, these biweekly surveys provide distribution and abundance data that could indicate whether white perch were displaced as a result of the storm events and the high discharge rates that occurred in the Hudson River^24^. An increase in abundance in the lower estuary after the storms would indicate that the fish had been displaced downriver.

## Results

### Environmental conditions

The annual mean water level in the Hudson River at Albany, New York (NY), in 2010 was 0.65 m and the mean water level in August 2010 was 1.98 m, which were similar to the averages for the decade 2002–2012 (mean ± s.d.: Annual 0.66 ± 0.08 m; August 1.97 ± 0.38 m). The water temperature at this site was also close to the average for the decade. The mean temperature in 2010 was 12.60 °C (Annual mean for 2002–2012: 12.46 ± 1.76 °C) and the mean in August 2010 was 24.74 °C (August monthly mean for 2002–2012: 24.84 ± 1.46 °C). The year 2010 was therefore considered a suitable control in our study based on the environmental conditions in the Hudson River being close to average.

**Figure 1:**
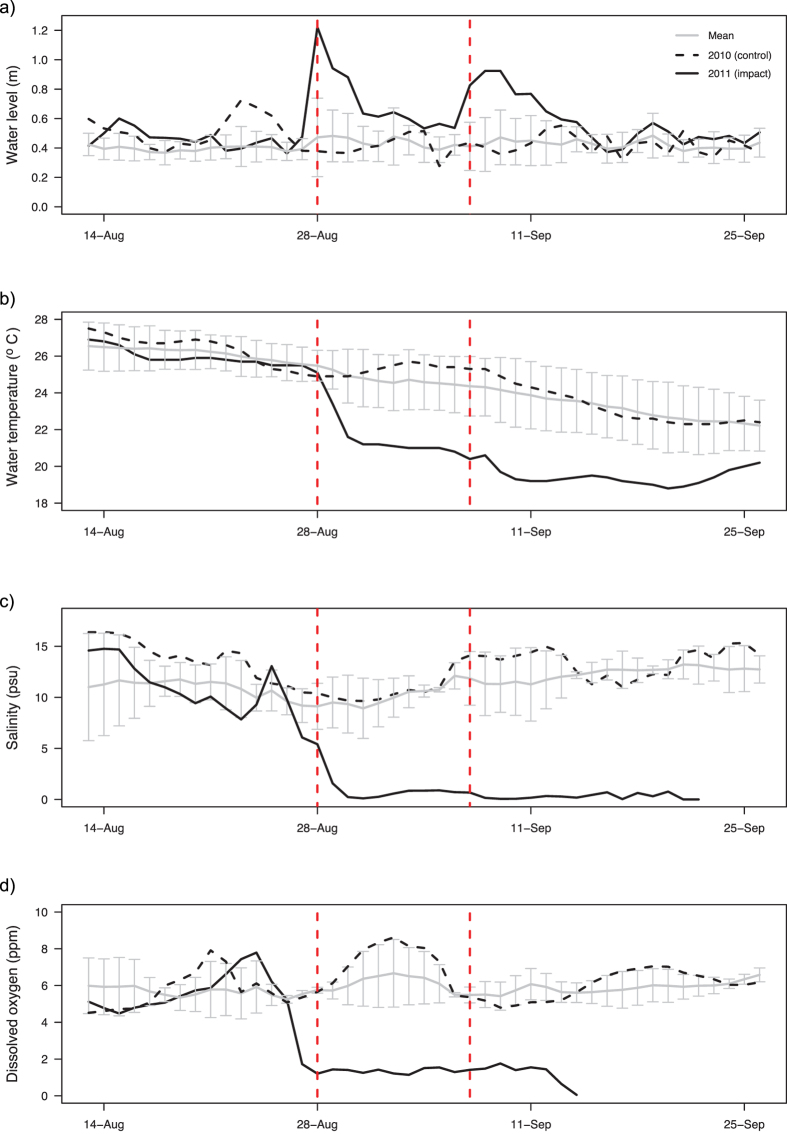
Environmental conditions during August to September in the Lower Hudson River Estuary in 2010 and 2011. Water quality parameters (**a**), water level, (**b**), water temperature, (**c**), salinity, and (**d**), dissolved oxygen. The mean (±SD) daily values are for the period 2002–2012 for water level and water temperature, and for the period 2008–2011 for salinity and dissolved oxygen. The vertical dashed red lines indicate the timing of the Tropical Storms Irene and Lee in 2011.

In 2011, much more extreme conditions in the Hudson River occurred as a result of the two tropical storms in late August and early September. The water level in the Hudson River rose rapidly because of the rainfall from the storms. It was significantly higher in 2011 during the storms in the upper estuary at Albany, NY, compared to preceding levels and those for the corresponding period in 2010 (F_2,83_ = 20.2, *p* < 0.001). The water temperature also decreased during the storm events and was significantly lower compared to the same time period in 2010 (F_2,83_ = 16.9, *p* < 0.001). Data on dissolved oxygen and salinity were not available for this site in 2010, but increases in dissolved oxygen and decreases in salinity were observed following each storm event in 2011 (Supplementary Figure S1). In the lower estuary, higher water levels were similarly recorded during the storms compared to preceding levels or those in 2010 (F_2,83_ = 28.4, *p* < 0.001), although the change in water level was not as great as in the upper estuary (Fig. 1 and Supplementary Figure S1). There were significant decreases in water temperature (F_2,83_ = 68.1, *p* < 0.001), salinity (F_1,55_ = 66.4, *p* < 0.001) and dissolved oxygen (F_1,55_ = 68.0, *p* < 0.001) in the lower estuary (Fig. 1).

The monthly mean discharge rate measured at the gauge at Green Island, NY, was 269 m^3^ s^−1^ in August and 263 m^3^ s^−1^ in September (averaged over the period 2002–2012). On 29^th^ August 2011 following Tropical Storm Irene, the daily mean discharge rate at this station reached 4,471 m^3^ s^−1^. At a cross-sectional channel area of 2,212 m^2^ (at stage 20)^25^, the water flow velocity would be up to 2.0 m s^−1^. After the Tropical Storm Lee, the daily mean discharge rate reached 3,028 m^3^ s^−1^ on 8^th^ September 2011 (estimated water velocity 1.4 m s^−1^).

### Striped bass movements

**Figure 2:**
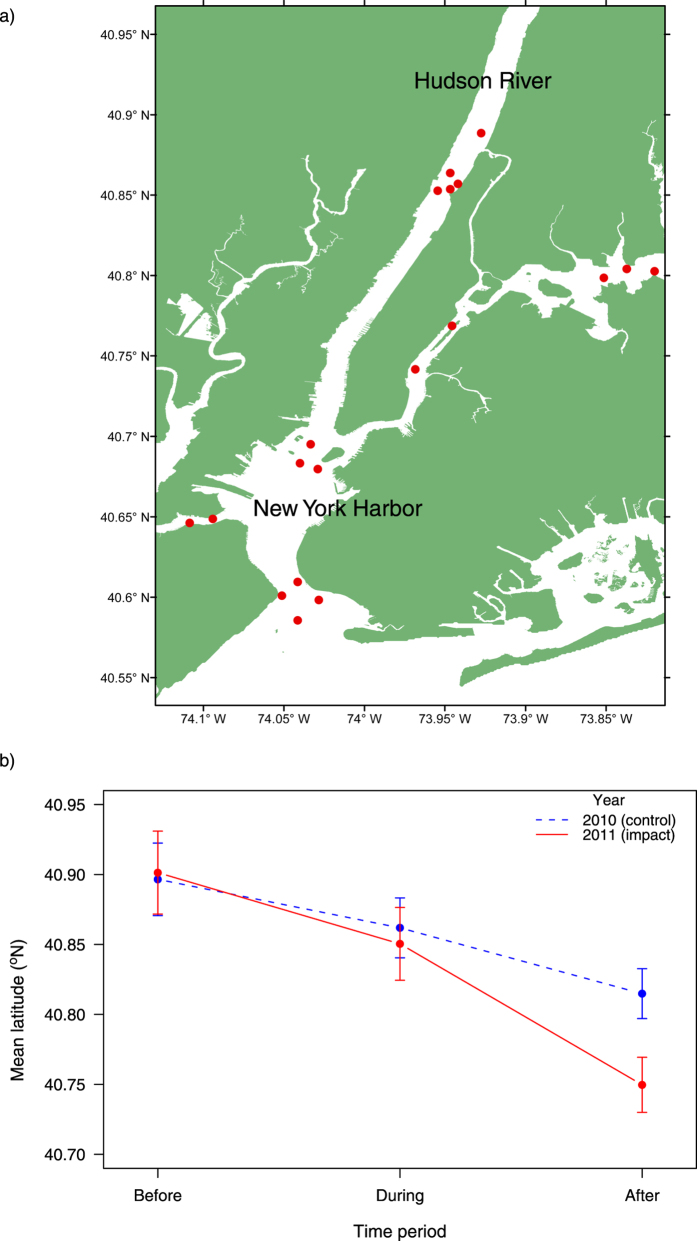
Coastal evacuation of fish in response to the storm events. (**a**) Map of Hudson River Estuary with acoustic receiver locations (red circles). The map was created in ESRI ArcMap version 9.3.1 (http://desktop.arcgis.com/en/arcmap). (**b**) Mean (±SE) latitude of tagged striped bass in the time period before (13^th^–27^th^ August), during (28^th^ August–11^th^ September), and after (12^th^–26^th^ September) the storms in 2011 and for the control year 2010.

Individual fish were tagged in October 2009 and May 2010 and acoustically tracked for a mean of 708 days (range = 160–816 days, individual fish n = 43). We identified a significant interaction between the time period (before-during-after) and event (control-impact) for the location of the fish indicating that displacement occurred after the storms with movement southwards out of the Hudson River Estuary and into the coastal environment (Supplementary Table S1). Although striped bass also moved southwards in the autumn of 2010 resulting in a decrease in mean latitude from late August to mid-September, this pattern was much more pronounced in the year of the storms (Fig. 2). When the analysis was repeated for the LEC subset only, individuals demonstrated a more dramatic southwards movement both during and after the storms in 2011 compared to the corresponding time period in 2010 (Supplementary Table S2 and Figure S2).

**Figure 3:**
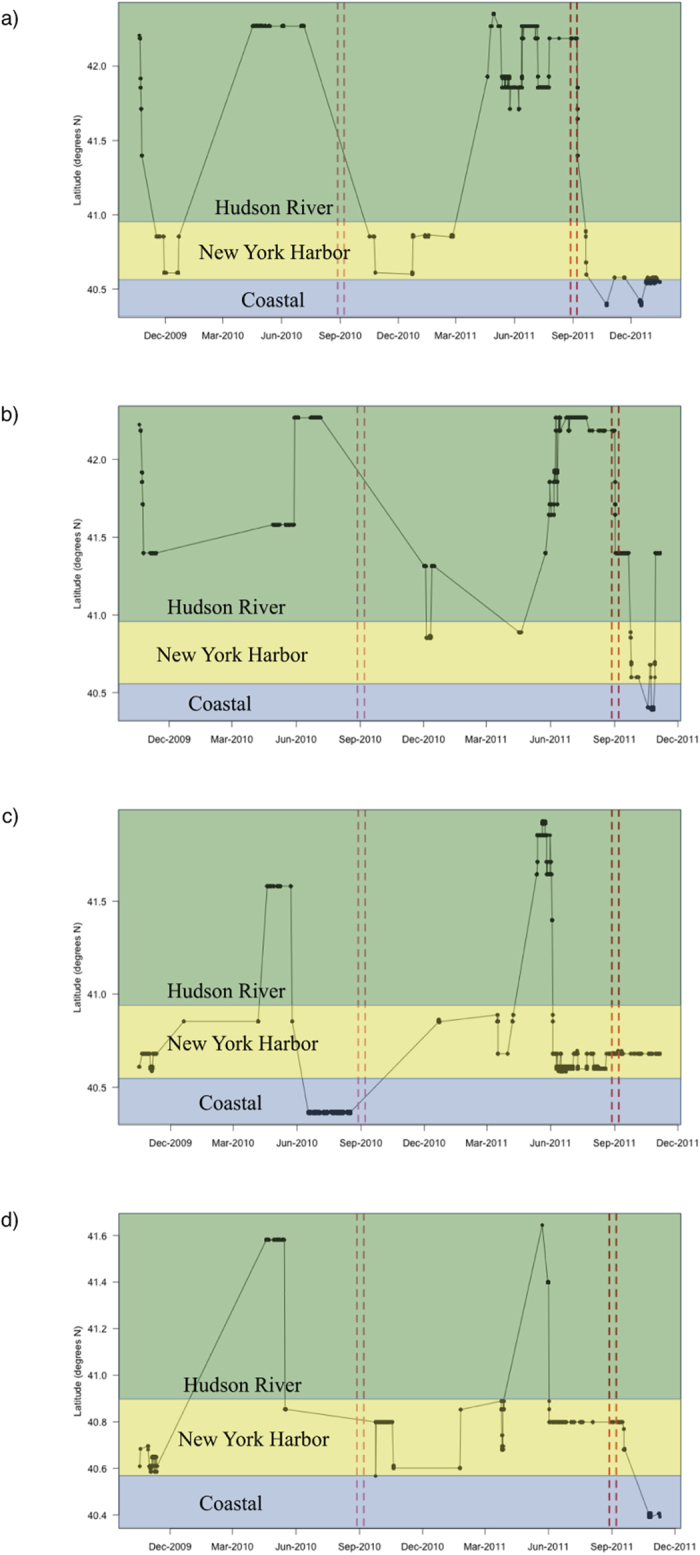
Individual movements of acoustically tagged striped bass in the Hudson River Estuary. Latitude of acoustic detections for the Upper Estuary Contingent (UEC) individuals (**a**), ID 3 and (**b**), ID 5, and for the Lower Estuary Contingent (LEC) individuals (**c**), ID 16 and (**d**), ID 36. The black circles represent dates of receiver detections, the bold vertical red lines indicate the timing of the Tropical Storms Irene and Lee in 2011, and the pale vertical red lines are the same time periods in 2010 for comparison.

Examination of the movement pathways indicated that initial location played a role in the response of striped bass. Fish that were in the Hudson River before the storms tended to move rapidly downstream and into the coastal environment during the storms, whereas those in the harbour either remained there or moved into the coastal environment and headed southwards (Fig. 3). In particular, three fish were detected at the same or nearby (within 2 km) receiver sites during the storm period in 2011 (n = 2 and 1 from the LEC and UEC respectively). The UEC fish (Study ID 1) was acoustically detected in the two-week period before and after the storms in the Hudson River near Catskill, NY, a site 184 river km from the mouth of the river. The two LEC fish (Study IDs 16 and 29) were detected almost daily (and often with multiple detections per day) during the entire before-during-after period of the storms at the three receiver sites in the northern part of New York Harbor (Fig. 3c). The UEC fish had a total length of 0.48 m (mean UEC tagged fish length = 0.49 m) and the LEC fish were 0.59 m and 0.71 m in length (mean LEC tagged fish length = 0.61 m). The water flow velocity of 2.0 m s^−1^ after storm Irene would correspond to a swimming speed of 4.2, 3.4 and 2.8 body lengths s^−1^ respectively for these fish.

**Figure 4:**
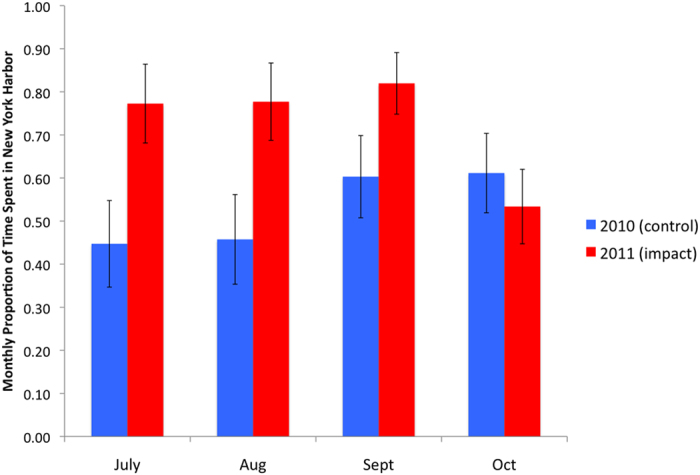
Residency time in New York Harbor in relation to the storm events. Monthly proportion of days spent in New York Harbor (±SE) by tagged striped bass from the Lower Estuary Contingent (LEC) in July to October of the control year 2010 and the year of the storms 2011.

We also analysed the effect of the storms on the residency time of fish within New York Harbor. The tagged striped bass spent significantly more time in the harbour in 2011 compared to 2010 (Supplementary Table S3). Contingent membership significantly influenced harbour occupancy, with a greater number of days spent in the harbour by the LEC than the UEC fish. There was no significant interaction between the time period and year variables indicating that the amount of time spent in New York Harbor did not significantly change during the period of the storms. Examination of the movement pathways indicated that changes in residency in the harbour occurred over longer time-scales than the two week before-during-after periods. We therefore also tested whether there was any difference in the proportion of days per month spent in the harbour from July to October in 2010 and 2011 (Supplementary Table S4). There was a significant interaction between the month of October and the year indicating that the amount of time spent in New York Harbor by tagged striped bass was significantly lower in October 2011, after the storms, than in 2010. This effect was even more pronounced in the LEC striped bass (Supplementary Table S5). In 2010, the proportion of time spent in the harbour was similar in July and August and increased in September, remaining high in October. In contrast, in 2011 there was a marginal increase in September, but a dramatic reduction in October (Fig. 4).

### White perch abundance

The adult standing stock of white perch in the Hudson River rose rapidly after the first storm event and was approximately twice the mean abundance for 1997–2013 for the four weeks (weeks 37–41) after the storms (Supplementary Figure S3). The geographic regions within the Hudson River Estuary did not have any data recorded for the sites upriver of Poughkeepsie (100–122 river km) for week 35 (30^th^ August 2011) following Storm Irene. However, the standing stock for older-than-yearling for the regions Battery (0–18 river km) and Yonkers (19–38 river km) in the lower Hudson River Estuary rose rapidly from 81,000 and 73,000 in week 35 to 380,000 and 831,000 in week 37 (12^th^ September 2011) respectively, and then declined again in weeks 39 (26^th^ September 2011) and 41 (10^th^ October 2011) (Supplementary Figure S3).

## Discussion

Evacuation behaviour may be triggered by environmental cues that indicate unsuitable conditions, inadequate resources, or unfavourable interactions with competitors and/or predators in the occupied habitat^26^. In our study, the behaviour was most likely initiated in response to the unsuitable conditions that arose in the Hudson River Estuary as a result of the extreme storm events. Tropical Storms Irene and Lee in 2011 caused heavy rainfall and major flooding in New York^24^ that resulted in high freshwater discharges and a rapid decline in water temperature. The strong water flows may have displaced white perch downriver into the lower estuary. It may also have potentially displaced striped bass out of the Hudson River and New York Harbor where the less suitable conditions, particularly the lower dissolved oxygen^27^, may have triggered the behaviour to evacuate into the coastal environment.

The detection of striped bass fully evacuating the Hudson River, rapidly moving downstream into the coastal environment, is noteworthy as a particularly unusual behaviour for the UEC fish, and suggests that the high discharge within the narrower river portion may have been sufficient to displace them. The extended movement into the coastal environment and even farther south than recorded in the previous year could also indicate evidence of initiation of the migratory syndrome, where movement behaviour continues despite resource-based stimuli that ordinarily stop further movement^18^.

Striped bass is a relatively large, long-lived anadromous species. Although many of the tagged fish had left the estuary and moved into coastal waters, there were three tagged fish that were detected throughout the storms and maintained their position in the Hudson River and New York Harbor. A swimming speed of 2.8–4.2 body lengths s^−1^ would have been necessary to swim against the peak daily mean water velocity. Speeds of up to 3.7 body lengths s^−1^ have been recorded over a distance of 5 km, but generally striped bass move at slower rates^20^. The maximum sustainable swimming speed recorded for striped bass is 2.9–3.3 body lengths s^−1^ during a 30–45 minute swimming bout^28^. It therefore seems unlikely the fish were continuously swimming at high speed during the period of the storms, which lasted several days^24^. They may have instead sought refuge within the estuary. Small-scale movements to refuge locations with cover or lower velocities has been observed in stream-dwelling fish during high discharge events^17^.

Contingent membership significantly influenced occupancy within New York Harbor, with a greater number of days spent in the harbour by the striped bass LEC than the UEC fish, which is consistent with what is known about these contingents’ movements^19^,^20^. The southward movement during the fall and winter in 2010 is also consistent with previous studies on their seasonal movements within the Hudson River Estuary^20^ and Chesapeake Bay^29^. However, the increased occurrence in the harbour in September followed by the rapid decline in October 2011 compared to 2010 supports the hypothesis that lower estuarine striped bass were displaced southwards by the storms into the harbour and had moved into the coastal environment by October.

White perch are generally most abundant in the upper estuary and more sparsely distributed in the lower estuary^30^. As expected, the abundance of white perch was relatively low in the lower Hudson River Estuary prior to the storms in 2011. However, there was a rapid increase in abundance of white perch in the lower Hudson River Estuary after the storms. The standing stock for older-than-yearling fish was 11 times greater in the week after the storms at Yonkers (19–38 river km) than it had been in the previous bi-weekly survey. This suggests that white perch moved out of the streams and upriver segments, with a downriver shift in their distribution after the storm, similar to that observed in striped bass. There was also an overall increase in abundance of white perch in the Hudson River Estuary for the four weeks after the storms indicating movement out of the streams and nontidal river segments.

A few of the tags transmitted long enough to enable the detection of three striped bass returning to New York Harbor during late October and November for short periods after the storm. This was then followed by a return to the harbour for the remainder of the winter and in some instances a continued movement northwards up the Hudson River. Seasonal site fidelity is well known for striped bass^20^, and these excursions may have served to re-establish regular movement patterns. Alternatively, this behavior could represent exploratory movements. Such movements would be expected to be sustained until suitable conditions are perceived^26^.

Although many of the striped bass LEC fish remained in coastal waters, two of the UEC fish for which we had longer tracking durations returned to the harbour and eventually to the Hudson River indicating the response to the storms lasted only a few months. However, if extreme events become more frequent in the future such changes could potentially become more severe or longer term. The timing of the extreme events is also likely to affect the strength and duration of the response and consequent impact on the fish community and ecosystem^31^,^32^,^33^. As extreme events become more frequent, telemetry will provide a valuable tool for linking event-scale forcing to migration responses. The estuaries and harbours that form important habitat for fish species are likely to experience more extreme conditions in the future as a result of climate change. Responses of fish species to extreme weather events should be considered when planning management strategies to ensure efforts are appropriately targeted to maintain key population segments and critical evacuation routes.

## Methods

### Ethics statement

This research was approved by the Institutional Animal Care and Use Committees (IACUC) of the University of Maryland Center for Environmental Science (Research Protocol No. 09C02) and was carried out in accordance with the approved guidelines.

### Environmental data

Water level and water temperature data were obtained from the U.S. Geological Survey (USGS) daily, monthly and annual statistics (available at: http://waterdata.usgs.gov/usa/nwis/dvstat/?referred_module=sw) for the station in the Hudson River at Albany, NY (USGS Station 01359139 at 42.646°N, 73.748°W), and in the lower estuary at the south dock at West Point, NY (USGS Station 01374019 at 41.386°N, 73.955°W) for the period 2002 to 2012. Salinity and dissolved oxygen data were obtained from the Hudson River Environmental Conditions Observing System (HRECOS, www.hrecos.org, accessed 20^th^ April 2016) for the Port of Albany Hydrologic Station (42.620°N, 73.759°W) and the lower estuary George Washington Bridge Station (40.852°N, 73.959°W). These data were recorded every 15 minutes and a daily mean was calculated. Data were available for 2008 to 2011 from the George Washington Bridge Station, but data collection only began on 4^th^ January 2011 at the Port of Albany Hydrologic Station. A before-during-after-control-impact design was applied with a repeated measures analysis of variance (ANOVA) to determine whether there were changes in water quality as a result of the storms. The time period was defined as the two weeks before (13^th^–27^th^ August), during (28^th^ August–11^th^ September), and after (12^th^–26^th^ September) the Tropical Storms Irene and Lee occurred in New York, USA. Sensor malfunctions as a result of the storms led to some missing data for salinity and dissolved oxygen. Only the before and during periods in the two years were therefore compared for these parameters.

Water discharge rates were obtained for the station in the Hudson River at Green Island, NY (USGS Station 01358000 at 42.752°N, 73.689°W) from the U.S. Geological Survey (USGS) daily and monthly statistics (available at: http://nwis.waterdata.usgs.gov/ny/nwis/inventory/?site_no=01358000&agency_cd=USGS). The water flow velocity was calculated by dividing the discharge rate by the cross-sectional area of the channel. The channel area at this site corresponding to the highest available stage value was used (stage 20)^25^.

### Striped bass acoustic telemetry data

In October 2009 to May 2010, 75 striped bass were tagged with individually coded ultrasonic transmitters (Vemco V16-4H-R64k; 67 mm, 10 g, 2.5 year expected battery life) in the Hudson River and New York Harbor^19^. These fish ranged in size approximately 400–1000 mm total length, and included three contingents called the upper estuary (UEC), lower estuary (LEC), and ocean contingents (OC)^20^,^21^, which were assigned on the basis of the location and season of capture^19^,^34^. Fifteen acoustic receivers were moored in and around New York Harbor in a “gates” design^20^ that allowed all tagged individuals to be monitored when they passed through any of the channels to the harbour. There were also additional receivers within the Hudson River and surrounding coastal environment (Fig. 2a). None of the tagged fish from the OC were present in the Hudson River or New York Harbor during the autumn of 2010 or 2011, and they were therefore not included in the analysis.

### Striped bass data analysis

We used the prior year, 2010, as our baseline control and compared it to the anomalous year with the extreme events, 2011. This is necessary as there is likely to be non-event related seasonal variation in striped bass use of the area that could act as a confounding factor if it is not taken into account. We determined if there were any changes in the location or behaviour of striped bass resulting from these events using a generalized linear mixed model (GLMM) with a before-during-after-control-impact design. The same time periods were used as in the analysis of the environmental data.

In the first model, our response variable was the latitude of the striped bass locations. The explanatory variables were the time period (before, during, after) and year (control, impact) and the interaction between these two categorical variables. We also included the contingent the fish belonged to as a covariate to account for seasonal differences in latitude that are generally found amongst the contingents. We calculated the mean daily location for each individual and interpolated daily positions between receiver sites to reconstruct the movement paths. This resulted in a daily latitude value for every day during the entire time period of interest for each individual. We excluded any movement paths that did not occur within the Hudson River Estuary or New York Harbor during the period of interest or that ceased transmitting before 26^th^ September 2011, which was the end of the study period. This resulted in a sample size of 22 individuals movement paths (n = 17 and 5 from the LEC and UEC respectively). There were 90 daily latitude values for each individual resulting in a total of 1,980 observations in the analysis. The track section in August to September each year was nested within the individual as a random effect in the GLMM to account for serial correlation in the movements of each individual tagged fish. We also repeated the analysis for the LEC fish only because of the larger sample size.

In the second model, we examined changes in residency patterns within New York Harbor. We used the same approach as in the previous model, but our response variable was the proportion of days per time period that fish were present in New York Harbor. We used the daily interpolated positions to include when the fish were within New York Harbor as well as detected by the acoustic receivers at its boundaries. We defined the New York Harbor area as within 1 km of our boundary acoustic receivers based on their expected detection range (Latitude: 40.575 to 40.898°N, Longitude: 73.810 to 74.120°W). Examination of the tracks indicated that changes in occurrence of striped bass in New York Harbor may have occurred over a longer time-scale and we therefore also tested whether there was any significant difference in the proportion of days per month from July to October between the control year 2010 and the year of the storms 2011.

### White perch data analysis

Adult white perch standing stock estimates were obtained for the period 1997–2013^35^. A mean standing stock for the Hudson River was calculated and compared to that for 2011 for the weeks 27 (5^th^ July 2011) to 47 (28^th^ November 2011), which included the storm events. The standing stock of older-than-yearling white perch was also compared for this period within specific geographic regions^35^.

## Additional Information

**How to cite this article**: Bailey, H. and Secor, D. H. Coastal evacuations by fish during extreme weather events. *Sci. Rep.* **6**, 30280; doi: 10.1038/srep30280 (2016).

## Supplementary Material

Supplementary Information
