# Supplementary material for: Coastal evacuations by fish during extreme weather events
Source: Sci Rep. 2016 Jul 26;6:30280. doi: 10.1038/srep30280 (PMC4960606; doi:10.1038/srep30280)
Supplement: Supplementary Information [file srep30280-s1.pdf]

Supplementary Information

Coastal evacuations by fish during extreme weather events

Helen Bailey and David H. Secor

Table S1: Results of the generalized linear mixed model (GLMM) for the location (latitude) of the tagged striped bass in relation to the categorical variables time period ("Before" given as the reference level) and Year (2010 given as the reference level) and their interaction. Contingent (Upper Estuary Contingent (UEC) given as the reference level) was also included as a covariate. An asterisk indicates where a P-value is less than 0.05 and is considered statistically significant.

**Factor**	**Estimate**	**SE**	**P-value**
Intercept	41.458	0.113	<0.001*
Contingent: LEC	-0.727	0.123	<0.001*
Time Period: During	-0.035	0.010	<0.001*
After	-0.082	0.010	<0.001*
Year: 2011	0.005	0.068	0.945
Interaction: During x 2011	-0.016	0.014	0.252
After x 2011	-0.070	0.014	<0.001*

Table S2: Results of the generalized linear mixed model (GLMM) for the location (latitude) of the Lower Estuary Contingent (LEC) tagged striped bass in relation to the categorical variables time period ("Before" given as the reference level) and Year (2010 given as the reference level) and their interaction. An asterisk indicates where a P-value is less than 0.05 and is considered statistically significant.

**Factor**	**Estimate**	**SE**	**P-value**
Intercept	40.699	0.041	<0.001*
Time Period: During	-0.004	0.006	0.476
After	-0.026	0.006	<0.001*
Year: 2011	0.006	0.058	0.921
Interaction: During x 2011	-0.023	0.008	0.004*
After x 2011	-0.044	0.008	<0.001*

Table S3: Results of the generalized linear mixed model (GLMM) for the proportion of days per 2-week time period that tagged striped bass were present in New York Harbour in relation to the categorical variables time period ("Before" given as the reference level) and Year (2010 given as the reference level) and their interaction. Contingent (UEC given as the reference level) was also included as a covariate. An asterisk indicates where a P-value is less than 0.05 and is considered statistically significant.

**Factor**	**Estimate**	**SE**	**P-value**
Intercept	-5.407	1.999	0.007*
Contingent: LEC	6.126	1.978	0.002*
Time Period: During	1.612	1.047	0.124
After	1.836	1.069	0.086
Year: 2011	4.192	1.712	0.014*
Interaction: During x 2011	-1.194	1.701	0.483
After x 2011	-0.553	1.767	0.754

Table S4: Results of the generalized linear mixed model (GLMM) for the proportion of days per month that tagged striped bass were present in New York Harbour in relation to the categorical variables month ("July" given as the reference level) and Year (2010 given as the reference level) and their interaction. Contingent (UEC given as the reference level) was also included as a covariate. An asterisk indicates where a P-value is less than 0.05 and is considered statistically significant.

**Factor**	**Estimate**	**SE**	**P-value**
Intercept	-2.476	0.894	0.006*
Contingent: LEC	2.666	0.816	0.001*
Month: August	0.064	0.709	0.928
September	0.984	0.730	0.178
October	1.039	0.732	0.156
Year: 2011	2.268	0.888	0.011*
Interaction: August x 2011	-0.023	1.165	0.984
September x 2011	-0.511	1.208	0.672
October x 2011	-2.797	1.120	0.013*

Table S5: Results of the generalized linear mixed model (GLMM) for the proportion of days per month that the Lower Estuary Contingent (LEC) tagged striped bass were present in New York Harbour in relation to the categorical variables month ("July" given as the reference level) and Year (2010 given as the reference level) and their interaction. An asterisk indicates where a P-value is less than 0.05 and is considered statistically significant.

**Factor**	**Estimate**	**SE**	**P-value**
Intercept	0.205	0.636	0.747
Month: August	-0.021	0.759	0.978
September	1.225	0.821	0.136
October	1.014	0.803	0.207
Year: 2011	2.504	1.125	0.026*
Interaction: August x 2011	0.091	1.465	0.950
September x 2011	-0.768	1.575	0.626
October x 2011	-3.392	1.324	0.010*

a)

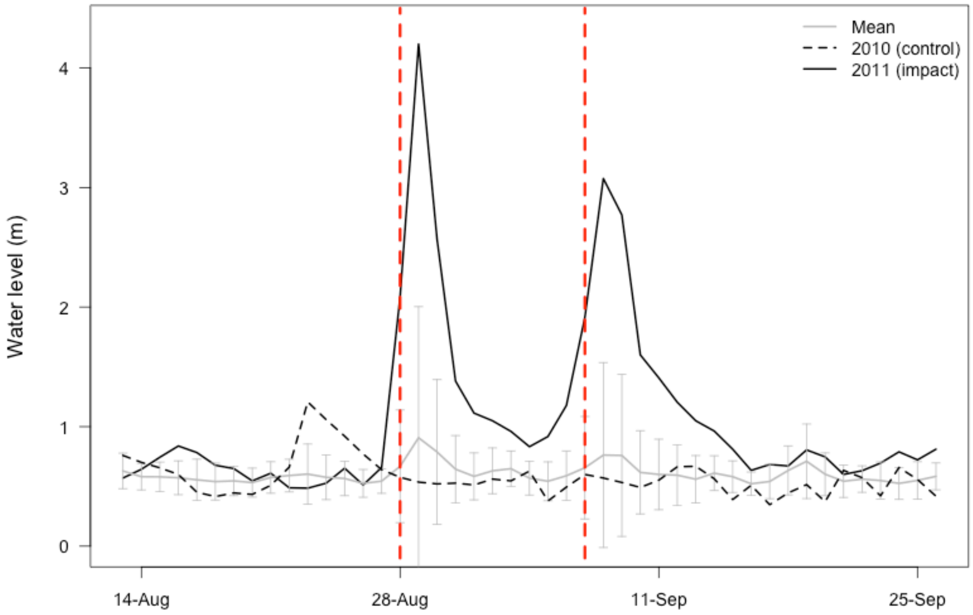
Mean
2010 (control)
2011 (impact)
Water level (m)
0
1
2
3
4
14-Aug
28-Aug
11-Sep
25-Sep

b)

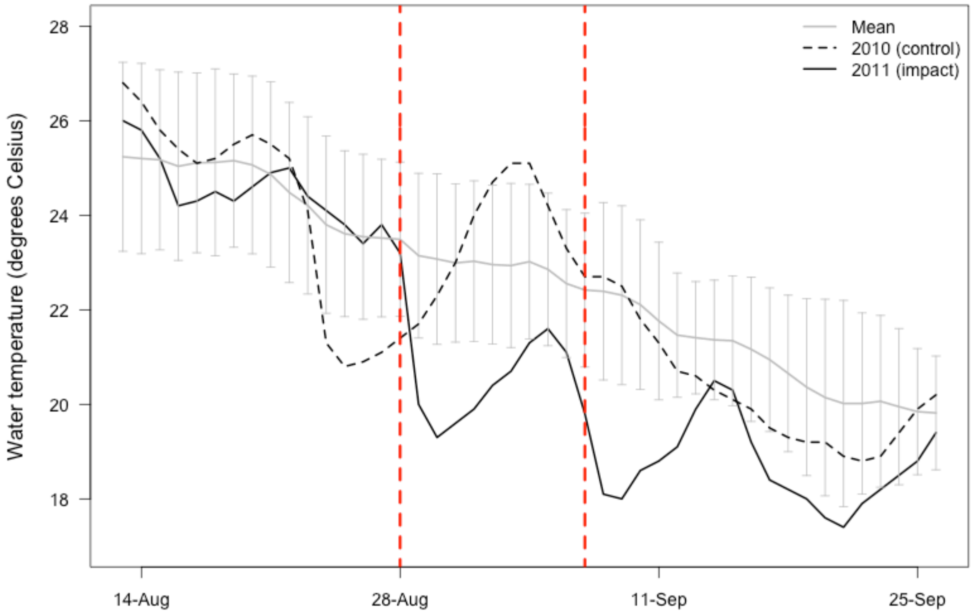
Mean
2010 (control)
2011 (impact)
Water temperature (degrees Celsius)
18
20
22
24
26
28
14-Aug
28-Aug
11-Sep
25-Sep

c)

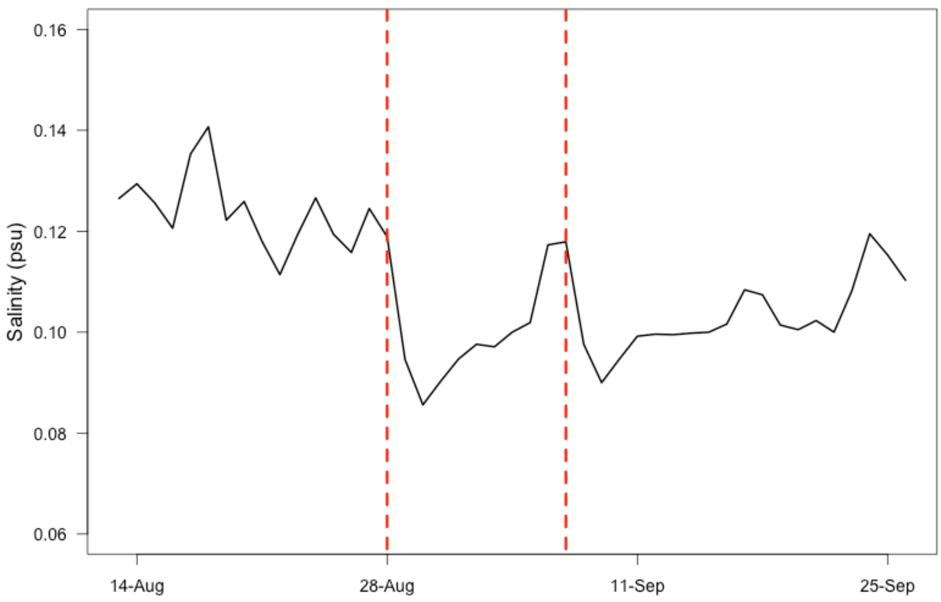

d)

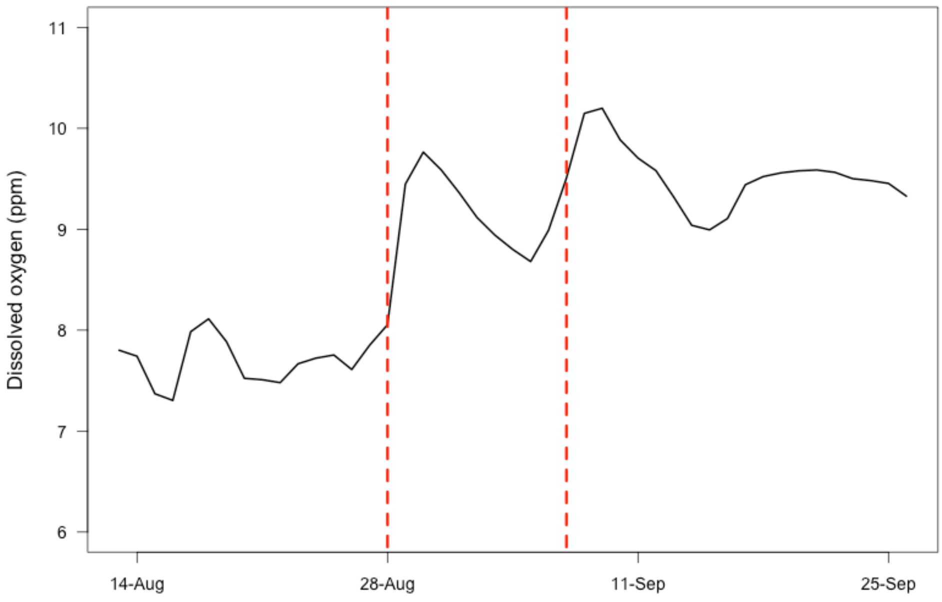

Figure S1: Environmental conditions in the Upper Hudson River Estuary. Water quality parameters in the Hudson River at Albany, NY, **a**, water level, **b**, water temperature, **c**, salinity, and **d**, dissolved oxygen. The mean (±SD) daily values are for the period 2002-2012 for water level and water temperature (data for salinity and dissolved oxygen were not available prior to 2011 for this station). The vertical dashed red lines indicate the timing of the Tropical Storms Irene and Lee in 2011.

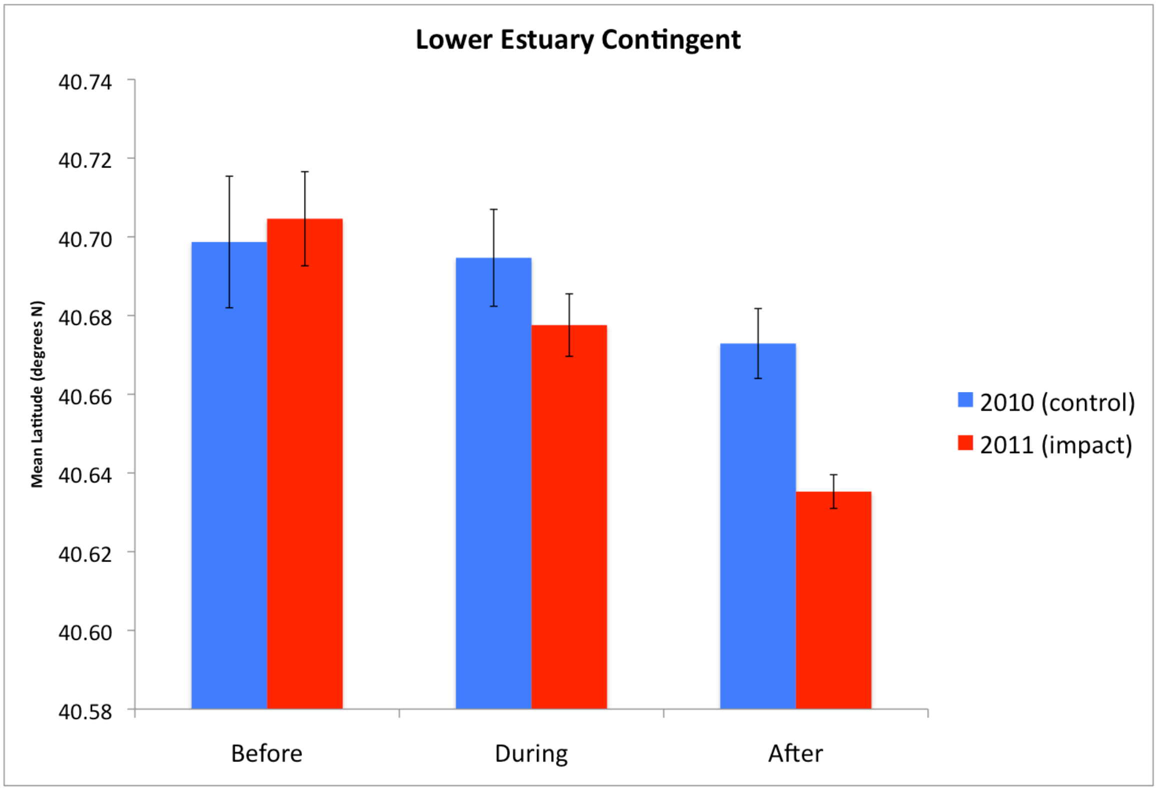

Figure S2: Movement of fish in response to the storm events. Mean (±SE) latitude of the Lower Estuary Contingent (LEC) striped bass in the time period before (13th - 27th August), during (28th August – 11th September), and after (12th-26th September) the storms in 2011 and for the control year 2010.

a)

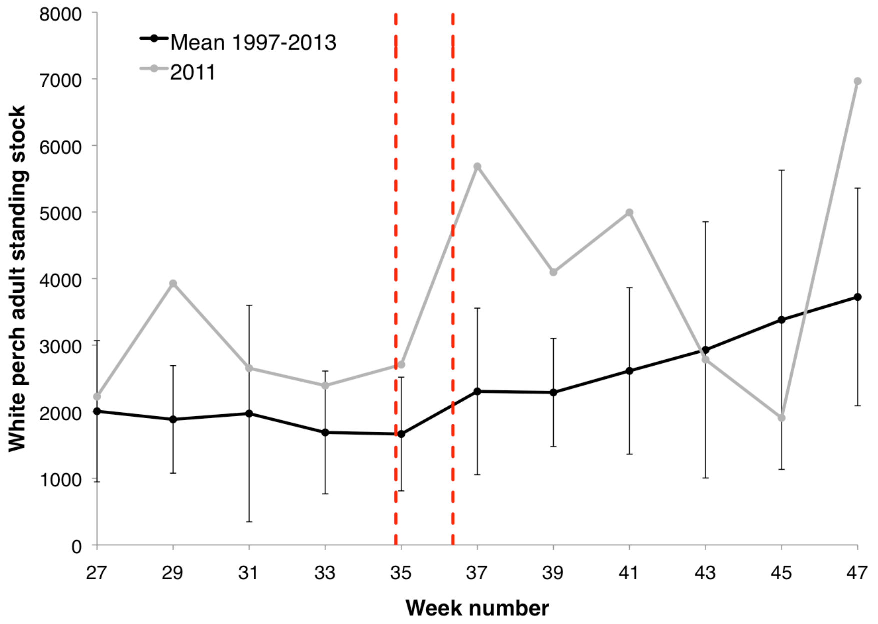

b)

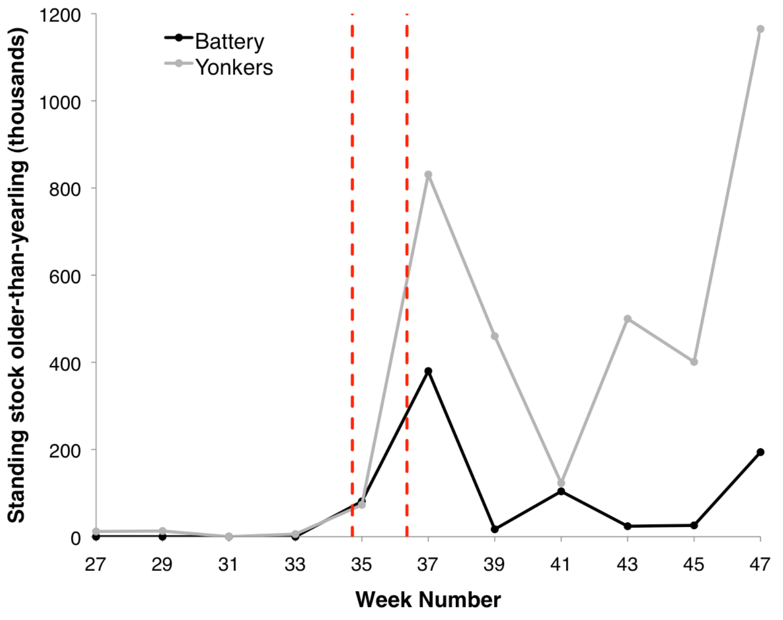

Figure S3: Abundance of white perch during the storm events. a) Adult standing stock of white perch in the Hudson River in 2011 and the mean for 1997-2013 (±SD), and b) Standing stock of older-than-yearling white perch in the Battery and Yonkers region in the lower Hudson River. The vertical dashed red lines indicate the timing of the Tropical Storms Irene and Lee in 2011.
